# The effect of nightly use of 150 mg cannabidiol on daytime neurocognitive performance in primary insomnia: a randomized controlled pilot trial

**DOI:** 10.1007/s00213-024-06674-x

**Published:** 2024-08-17

**Authors:** Andrea J. Narayan, Amie C. Hayley, Sarah Rose, Lauren Di Natale, Luke A. Downey

**Affiliations:** 1 Centre for Mental Health & Brain Sciences, Swinburne University of Technology, Hawthorn, 3122 Australia; 2 Institute for Breathing and Sleep, Austin Hospital, Melbourne, Australia

**Keywords:** Cannabis, Cognitive, Performance, Insomnia, Cannabidiol, CBD

## Abstract

**Rationale:**

Cannabidiol (CBD) is increasingly used as a sleep aid for insomnia; yet neurocognitive and subjective state effects following daily therapeutic use are unclear.

**Objectives:**

To measure the effect of daily CBD use on neurocognitive performance and daily subjective mood in a population with primary insomnia.

**Methods:**

This study used a randomized, placebo-controlled, parallel design incorporating a single-blind placebo run-in week followed by a two-week double-blind dosing period, during which participants consumed 150 mg CBD (*N* = 15) or placebo (*N* = 15) sublingually 60-minutes daily before bed. Attention, executive function, reasoning, information processing, working and episodic memory were assessed using the CogPro system at the beginning of the placebo run-in, after 1-week and 2-weeks of dosing. Subjective states using visual analogue scales and side effects were recorded daily.

**Results:**

Cognitive performance was unaffected by nightly CBD supplementation (all *p* > 0.05). From baseline to trial conclusion, those receiving CBD reported greater experience of calmness, clear-headedness, coordination and were more likely to report side-effects of dry mouth relative to placebo (all *p* < 0.05).

**Conclusions:**

Relative to placebo, daytime cognitive functioning following nightly supplementation as a therapeutic aid for primary insomnia was preserved under trial conditions. Results suggested an overall favourable safety profile, with larger controlled trials and thorough analyses of varying insomnia phenotypes necessary to corroborate these findings.

**Supplementary Information:**

The online version contains supplementary material available at 10.1007/s00213-024-06674-x.

## Introduction

Sleep is necessary for neural connectivity and plasticity that forms the basis of acquiring, retaining and integrating new information (Claßen et al. 2022). Globally, as many as 20% of adults report insufficient or disturbed sleep at least every other night (Morin and Jarrin 2022) and up to 30% meet criteria for a clinical sleep disorder such as insomnia (Xu et al. 2019). Although insomnia is primarily recognized as a disorder of nocturnal sleep disturbance, impairments or distress in daytime functioning and areas of cognitive performance frequently co-occur, and, as such, are recognised as an important clinical feature of the disorder. These feature of insomnia contribute to its social and economic burden (Morin and Jarrin 2022), with an estimated 40% being at risk for developing co-morbid disorders (Ohayon 1997). First-line pharmacological treatments often employed for the alleviation of sleep loss in insomnia, such as benzodiazepines including temazepam, can produce recognisable negative residual effects on alertness, concentration and memory, in addition to decreased self-reported quality of life (Fitzgerald and Vietri 2015). These medications are thus not considered suitable for daily or long-term use, and may exacerbate pre-existing cognitive impairments (Fitzgerald and Vietri 2015). Therefore, it is important to explore efficacious and safe alternatives that may provide universal relief for both night and daytime symptoms in insomnia.

Medicinal cannabis has been gaining popularity as a broad-spectrum medicine for sleep, mood and a range of other clinical conditions (Bridgeman and Abazia 2017), with insomnia being the third most common reason for its prescription in Australia (Maddison et al. 2022). Commonly researched cannabinoids include the psychoactive delta-9-tetrahydrocannabinol (THC) and cannabidiol (CBD), the latter of which is known to be non-intoxicating whilst maintaining a broad spectrum of pharmacological action (Kocis and Vrana 2020). The limited number of studies explicitly exploring the effects of CBD-only treatments on sleep outcomes reported some potential for improvements in insomnia symptomology for doses between 18 mg-800 mg, with future studies necessary to corroborate findings and reach consensus on therapeutic guidelines including dose size, treatment route and period (Narayan et al. 2022; Suraev et al. 2020; Ranum et al. 2022). Furthermore, evidence suggested increased somnolence as a side effect of CBD drug-drug interactions specifically when co-administered with drugs for epilepsy (clobazam), depression (sertraline, tranylcypromine, phenelzine, and isocarboxazid) or opioids (morphine) (Balachandran et al. 2021). Conversely, alerting effects associated with CBD have been observed in healthy adults, typically when administered in combination with THC at higher doses (Nicholson et al. 2004). Together, this suggests that sleep-enhancing effects of CBD may be somewhat malleable and dependent on concomitant treatments, dose sizes, and resultantly may affect next day performance due to these side effects. Despite its frequent prescription as a sleep aid, the potential acute and residual effects of CBD on cognitive impairment often linked with insomnia (Fortier-Brochu et al. 2012; Wardle-Pinkston et al. 2019; Brownlow et al. 2020; Ballesio et al. 2019) remains largely unexplored.

Studies investigating the effects of CBD on next-day cognitive function reported limited positive effects or no effects and utilized CBD doses ranging from 15 mg to 1500 mg (Jones and Vlachou 2021; McCartney et al. 2022; Martin et al. 2019; Boggs et al. 2018; Hallak et al. 2010) alone or in combination with other cannabinoids including THC (Woelfl et al. 2020). Participants receiving CBD have demonstrated selective attention capacity and processing speed improvements (Hallak et al. 2010), with impairments in cognitive processing speed and attention tasks relative to THC only (Woelfl et al. 2020). Moreover, greater divided attention tracking errors were reported for 15 mg of CBD compared to 300 mg and 1500 mg doses without clarity on if doses impaired or improved performance (McCartney et al. 2022). Therefore, should CBD prove to effectively enhance sleep in insomnia without impairments to cognitive performance as discussed, it could potentially enhance overall day-time insomnia symptoms which are typically exacerbated by current treatments.

Given the increasing availability and use of cannabinoid medications as a therapeutic sleep aid, it is necessary to understand the potential consequential effects on daytime functioning, particularly in samples with insomnia, where impairment to cognitive functioning is a common secondary symptom. Therefore, this study aimed to explore the effect of nightly supplementation of the over-the-counter dose of CBD (150 mg) sanctioned for sale in Australia on neurocognitive performance in a sample of adults with self-reported moderate to severe primary insomnia over a 2-week dosing period and compared to placebo at baseline, after 1-week of daily dosing and after 2-weeks of daily dosing (end of the trial). Moreover, it aimed to assess daily subjective mood and side effects during the 3-week trial period. It is hypothesized that daily CBD intake would improve cognitive performance in primary insomnia in addition to subjective state effects compared to placebo with minimal side effects.

## Study design and methods

### Trial design

This study was a sub-analysis of data collected as part of a broader pilot trial assessing the effects of 150 mg of CBD on sleep outcomes [for results of the broader trial please see Narayan et al. (2024)]. It consisted of a single blind placebo run-in week followed by a 2 week, randomized, double blind, placebo controlled parallel arm design. The broader trial was prospectively registered with the Australia and New Zealand Clinical Trials Registry (ID: ACTRN12620000070932) and approved by the Swinburne University of Technology’s Human Research Ethics Committee (approval no. 20220392-9708) on January 20th, 2020. All participants provided verbal and written consent at pre-screening and the in-lab screening visit respectively with ongoing consent provided at each subsequent testing visit. This trial was conducted in accordance with Good Clinical Practice guidelines and the ethical standards of the Declaration of Helsinki. All visits were conducted at Swinburne University of Technology, with daily self-reported measures logged by participants at home.

### Telephone screening (Pre-screening), In-Lab screening visit (V0)

**Fig. 1:**
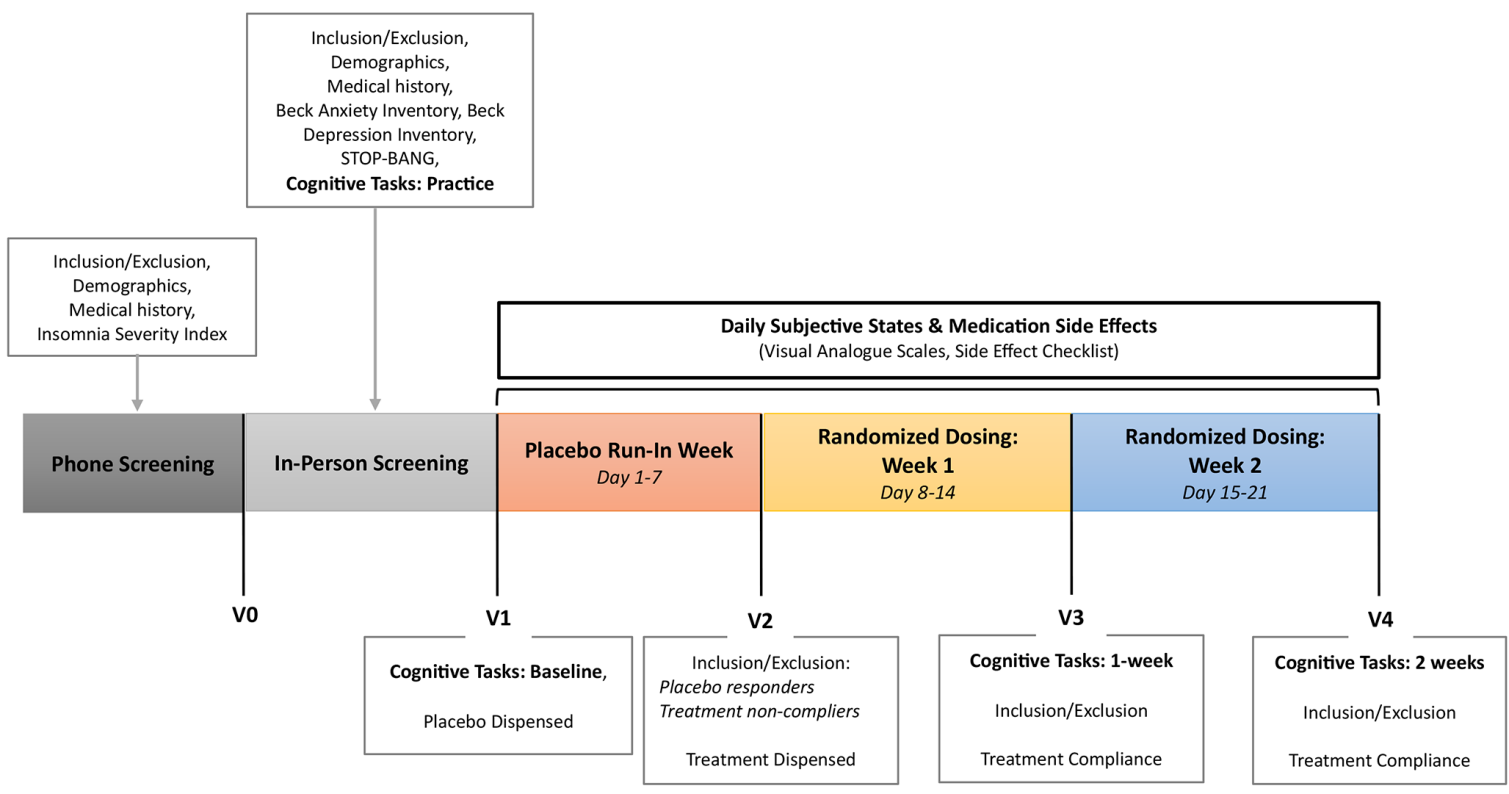
Study schedule. Study schedule, testing visits and measures at each trial phase

Those enrolled were pre-screened and excluded if they reported the presence of significant medical conditions (e.g., parasomnias, psychiatric or clinical conditions), were taking regular medication that may reasonably affect sleep (e.g., antidepressants, opioids, benzodiazepines), were engaged in shift work (self-report) or reported excessive daily caffeine consumption (> 400 mg caffeine/4 cups of coffee). Participants aged between 18 and 45 years who met the criteria and who reported ongoing moderate-severe insomnia symptom severity with no formal medical diagnosis [as determined by an Insomnia Severity Index (ISI) score of ≥ 15] were scheduled for the in-lab screening visit (V0) (see Fig. 1: Study Schedule). The in-lab screening visit (V0) identified and excluded those with probable mood disturbances [Beck Depression Inventory (BDI) score ≥ 20 (Beck et al. 1988); Beck Anxiety Inventory (BAI) score ≥ 16 (Beck et al. 1988)] and likely moderate to severe risk of obstructive sleep apnoea [STOP-BANG score ≥ 5 (Chung et al. 2012)] as per the exclusion criteria for the broader investigation. A clinical interview with a research nurse (and additional confirmation from a study physician) further screened for the existence of any physical or psychological conditions, medication or supplement use that may have been a contraindication to the investigational product. Once all eligibility criteria of the broader trial was confirmed, participants completed two practice rounds of all cognitive tasks.

### In-Lab testing visit - Placebo Run-In Week (V1 - V2)

Those meeting the described eligibility criteria at V0 began the 1-week placebo run-in period (V1), with placebo responders and treatment non-compliers being identified and excluded at the next in-lab visit, a week later (V2) (see Fig. 1: Study Schedule). Throughout the placebo run-in period, participants were instructed to continue with their normal daily routines, take their assigned treatment nightly (as outlined in *Study Treatment* section), and fill in a daily treatment logs, subjective states scales and side effects questionnaire using the pen and paper booklets provided, in addition to measuring daily objective sleep outcomes using GENEActiv wrist-mounted actigraphy watches (version 1.1, Activinsights, Kimbolton, United Kingdom). Data for the sleep quality criteria was collected through daily self-reported pen and paper sleep diary entries over the placebo run-in week. On conclusion of the V1 run-in week, those who met criteria for one or more of the sleep quality criteria of; subjective sleep efficiency (SE) (> 85%), sleep onset latency (SOL) (< 31 min) or wake after sleep onset (WASO) (< 31 min) were deemed placebo responders and excluded from further participation. Participants were also excluded if found to be treatment non-compliers (those with 20% or more missing doses as measured by treatment bottle weight), reported excessive use of treatment (> 15 ml used) or had > 20% of actigraphy-derived data missing. Once these criteria were satisfied, participants were randomized into treatment groups (either CBD or placebo, see below for treatment details) to begin the 2-week trial period. During this period, bottle weight was checked at during in-lab visits to ensure participants took treatments in accordance with daily self-reported treatment logs, with those being excluded for missing 20% or more doses, or found using excessively (> 15 ml used within the week).

### Study treatments

CBD and placebo treatments (corn oil only) were provided by Brains Bioceutical, UK and compounded into indistinguishable 30mL bottles of 100 mg/mL CBD treatments by Aspa pharmacy, Prahran, Melbourne and safely stored on at the testing centre at Swinburne University of Technology before dispensing. Participants received instructions to consume 1.5mL of the assigned treatment via the provided opaque 3 ml dosing syringe (equating to 150mL target treatment dose), 60 min before bed nightly. The dispensing pharmacy ensured active and placebo oils were indistinguishable in appearance and odour via participant visual examination and smell, with no alterations to the taste of either treatment. Placebo run-in treatment bottles were numbered 001 to 040 and double-blind treatments were numbered 041 to 080 and dispensed in numerical order. Both placebo and active treatment bottles were amber in colour. Staff independent of the trial upheld the key to treatment coding until the completion of data collection. Randomization software (Research Randomizer Software Version 4.0) was used to generate a random treatment order. Researchers remained blinded over the active dosing period until all participated had competed the trial. Numbered treatment bottles and a pre-determined order for dispensing was effectively kept by staff not involved in the trial to maintain blinding regardless of receiving placebo or CBD. Despite efforts to ensure the gold-standard, double-blind nature of the trial, blinding was not explicitly assessed throughout the 3-week period.

### Neurocognitive performance

Primary outcomes included cognitive performance measures within the domains of attention, concentration, and vigilance, working memory and executive control and episodic/declarative memory using computerized CogPro testing system (Ecog Pro Ltd., Bristol, UK) (Wesnes et al. 2017). Cognitive performance was measured at the start of the placebo run-in week (V1), 1-week after daily dosing (V3) and 2-weeks after daily dosing/trial end (V4) (see Fig. 1: Study Schedule). Due to treatments being taken prior to sleep, cognitive testing typically occurred over 8 h after the last treatment.

#### Simple and complex reaction time

The simple reaction time task measured the speed of simple motor responses to imminent and expected stimulus at unpredictable intervals. In addition to focus concentration, complex reaction time tasks included information processing to identify and select the appropriate response to presented stimulus. Recorded outcomes included simple reaction time median, complex reaction time mean [both in milliseconds (ms)] and accuracy (*%*). Simple reaction time mean scores were not available through the CogPro testing suite, therefore resulting in only median speed outcomes to be used in analysis.

#### Digit vigilance

Vigilance was measured by correctly detecting a target digit amongst a series of unpredictable digits, with a constantly displayed target removing the involvement of working memory. Recorded outcomes included the reaction time mean of the correct detections (ms) and its accuracy (%) in addition to error responses (false alarms).

#### Numeric working memory

The ability to hold numeric information and rapidly retrieve it was measured by showing a target series of 5 digits prior to the presentation of a series of 30 digits presented one by one for 1150ms at 50ms intervals. Participants were required to indicate, as quickly and accurately as possible using the keypad, whether or not the presented digit was part of the target series.

Outcomes included overall reaction time mean to correct responses for all stimuli (ms), reaction time means to original stimuli and new stimuli (ms) and the accuracy of responses to both new and old stimuli (%).

#### Spatial working memory

The spatial working memory task measured the ability to hold and retrieve information in working memory accurately and quickly. Participants were required to remember the spatial pattern of first shown prior to a series of ‘probe’ stimuli presented randomly, one at a time. The accuracy (%) and speed (ms) of correctly identifying a probe in the location of one of the original stimuli was used. Recorded outcomes included reaction time means to overall, original and new stimuli (ms) in addition to response accuracy to new and old stimuli (%) were recorded.

#### Immediate and delayed word recall

Individually, 15 words were presented on screen for 1500ms at a 500ms interval after which, participants typed as many words are they recalled within 60 s. Number of correctly recalled words, accuracy (%) and errors were recorded as outcomes.

Delayed recall was carried out after tests of attention and working memory, with participants typing as many words as they remembered from earlier. Correctly recalled words, accuracy (%) and errors were recorded.

#### Word recognition

Original words participants had seen previously, and distractor words, were individually presented in a random order. Participants responded as quickly and accurately as possible to whether they had or had not seen the presented word. Response accuracy for original and new stimuli (%) and reaction time means to overall stimuli, original and new stimuli (%) were recorded.

#### Picture/pattern recognition 1 and 2

Similar to word recognition, original pictures and distractor pictures were individually presented in a random order. Participants were required to respond as quickly and accurately as possible with accuracy (%) for original and new stimuli and reaction time means for overall, original and new stimuli responses (ms) recorded.

### Subjective measures

#### Subjective states [Visual analogue scales (VAS)] and self-reported side effects (daily log)

**Fig. 2:**
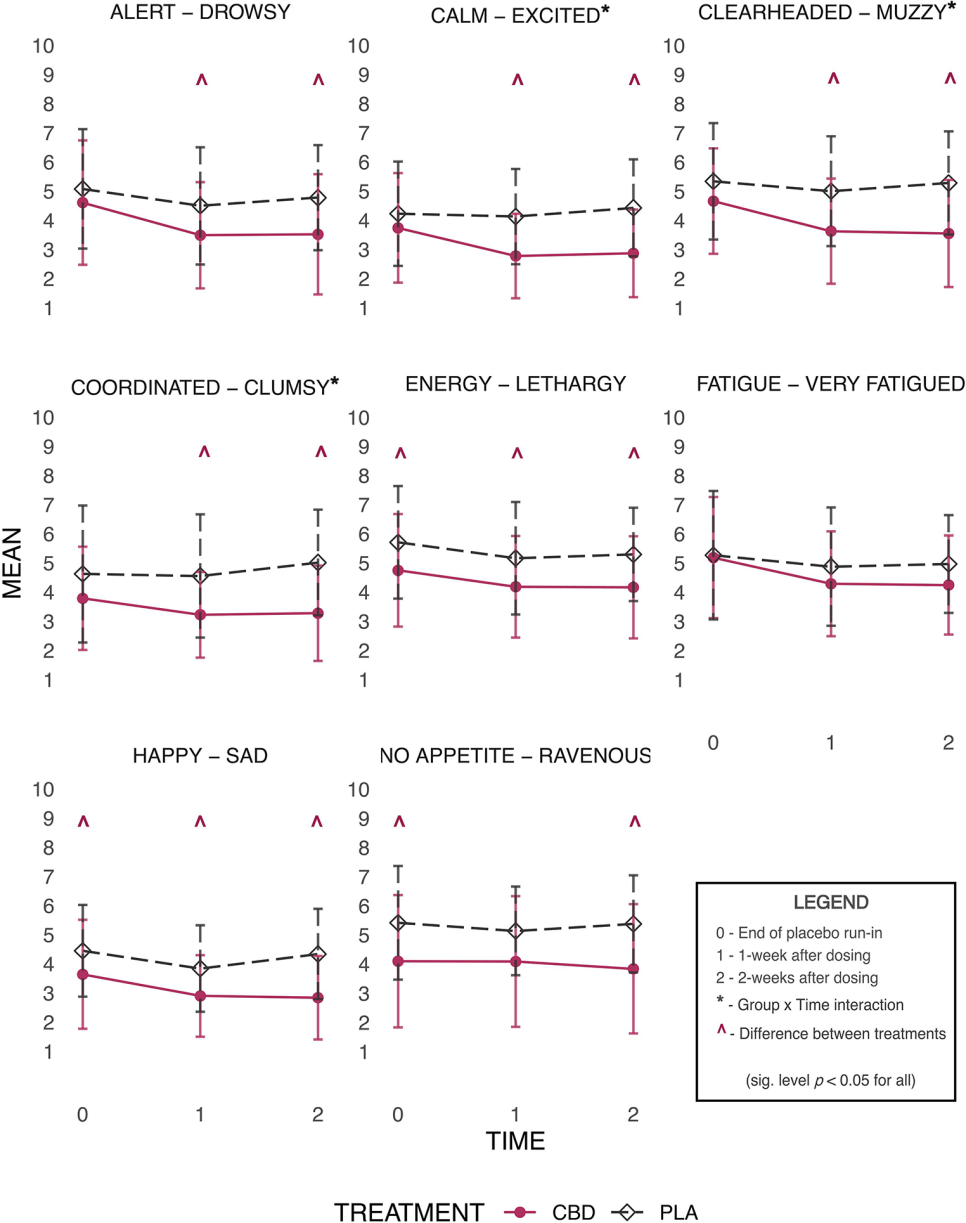
Subjective States Outcomes. Visual analogue scale outcomes - raw means with standard deviation error bars. *Note*: This panel of figures shows VAS scales for each subjective state measured across time where 0 = end of placebo run-in, 1 = after 1-week of dosing, and 2 = after 2-weeks of dosing. The symbol ^ denotes a statistically significant difference between treatments (*p* < 0.05). An asterix (*) denotes a significant Group × Time interaction effect (*p* < 0.05)

Daily subjective states were logged every morning using a 10-point VAS Likert scale provided in participant sleep diaries. Lower numbers corresponded to a positive effect and higher numbers corresponded to its opposite effect. Participants rated feelings of *alert-drowsy*,* calm-excited*,* clearheaded-muzzy*,* coordinated-clumsy*,* energy-lethargy*,* no appetite-ravenous*,* happy-sad* and *no fatigue-very fatigued* (Fig. 2). Secondary outcomes included daily self-reported side effects (present/not) of *nausea*,* dizziness*,* dry mouth*,* light headedness* and *diarrhoea* over the 3-week period (Online Resource 6).

#### Analytical plan

G*Power (version 3.1) was used to establish the necessary sample size to observe a small-medium effect size (Cohen’s *f =* 0.3) across a two way between-subjects design on reduction of ISI score (primary outcome). A medium effect size, 80% power and a 0.05 *p*-value required 23 completed participants. Thus, this trial intended for 15 completed participants per treatment group (*N* = 30).

Group differences in baseline demographic characteristics were analysed using independent t-tests for continuous variables and chi-square tests of independence for categorical variables. Separate linear mixed-effects model analyses (SPSS syntax: MIXED) with restricted maximum likelihood estimation were used to assess treatment-specific changes across time for each cognitive task outcomes at the start of placebo run-in (V1), 1-week after daily dosing (V3) and 2-weeks after daily dosing (V4). Daily subjective state VAS outcomes were physically collected at the end of each week of the study [end of the placebo run-in (V2), 1-week after daily dosing (V3) and 2-weeks after daily dosing (V4)]. Each daily rating for each scale were entered according to week and analysed using separated linear mixed-effect models. For cognitive task and VAS outcomes, either compound symmetry or autoregressive (AR1) likelihood ratio statistic was used, depending on best fit for variance structure for primary outcomes. For each model, group and time were entered as repeated variables. Group, time and the interaction of group and time were entered as fixed effects. Each model had participant ID entered as a subject grouping factor for random effects to account for both individual differences at baseline and responses to predictor variables for repeated measures over time (Brown 2021). Log and square root transformations were applied for residuals with non-normal distribution (as determined by a Shapiro-Wilk test with *p* < 0.05 and examination of distribution plots), with the original data being analysed if distribution remained non-normal after transformation (Schielzeth et al. 2020). When a main effect was observed, post-hoc paired t-tests with Bonferroni correction for multiple comparisons were examined. The average of original and new stimuli reaction time and accuracy were calculated and used in separate linear mixed effect model analyses for cognitive domains measuring these outcomes. All statistical analyses were performed using SPSS (version 29) with tests being two-tailed and statistical significance defined as *p* < 0.05.

## Results

Between February 2021 and September 2022, 810 registrants recruited from Swinburne trial databases, oral communications, physical posters and online ads (via social media) were sent the participant information and consent form, of which 88 passed pre-screening and 76 were excluded (see Online Resource 1). A further 18 participants withdrew due to scheduling conflicts, leaving 70 eligible participants attending the in-lab screening visit. Of this, 15 participants were excluded for BAI/BDI scores exceeding the cut-off, and three participants opted to discontinue participation with 52 remaining participants eligible to begin the placebo ruin-in week.

Prior to the start of the placebo run-in, eight participants withdrew from the trial, leaving 44 participants starting the placebo run-in week (V1) and being assessed at the end of the placebo run-in period (V2). At the end of the placebo-run in week (V2), placebo responders (*n* = 8) were identified and excluded, with 1 withdrawal due to scheduling conflicts and 1 adverse event of anxiety and paranoia. Eligible participants were then randomized to receive CBD (*n* = 18) or placebo (*n* = 16). At the end of 1-week week of dosing (V3), one participant in the CBD group reported side effects of ongoing restlessness resulting in their withdrawal from the trial, whilst another participant in the placebo group opted out for other reasons. Both adverse events resulting in withdrawals were mild and short-lived, with affected participants put in contact with any necessary services during debriefing. At 2-weeks after dosing, 32 participants had completed the trial. In accordance with a-priori trial registration, data of a final sample of *N* = 30 participants satisfying trial requirements were analyzed (Narayan et al. 2024). The randomization of extra participants (*n* = 4) was done to address potential late-stage attrition (*n* = 2) and any significant COVID-19 related delays that posed a risk of failure in recruitment targets being met prior to investigational product expiry (*n* = 2) (see Online Resource 1) (Narayan et al. 2024). Analyses conducted with *N* = 32 showed no significant changes to overall outcomes. Therefore, the outcomes reported are for *N* = 30, as reported in Narayan et al. (2024).

### Demographic outcomes (table 1)

**Table 1:** Demographics. Baseline demographic and clinical group characteristics and group comparison statistics (t-value or chi-square with degrees of freedom [χ2 (df)] and corresponding *p*-value

Baseline characteristic	CBD		Placebo		Full Sample		t-value χ2 (df)	*p*-value
	*n*	%	*n*	%	*n*	%		
Gender							**0.13 (1)**	0.72
Female	8	53	7	47	15	50		
Male	7	47	8	53	15	50		
Height *cm* Mean (SD)	173.77 (7.13)		175.74 (8.52)		174.76 (9.61)		0.55	0.29
Weight *kg* Mean (SD)	70.18 (19.24)		75.14 (17.33)		72.66 (18.47)		7.17	0.24
Age Mean (SD)	33.47 (7.13)		29.67 (6.00)		31.57 (6.84)		-1.53	0.14
Handedness							**2.15 (2)**	0.34
Left			2	13.3	2	6.7		
Right	14	93	12	80	26	86.7		
Ambidextrous	1	6.7	1	6.7	2	6.7		
Total years education Mean (SD)	17.4 (3.22)		15.70 (2.29)		16.55 (2.92)		-1.61	0.06
Highest educational level^1^							**0.42 (2)**	0.81
Secondary	2	13.3	3	20	5	16.7		
Tertiary	9	60	10	66.7	19	63.3		
Postgraduate	3	20	2	13.3	5	16.7		
Employment							**3.3 (3)**	0.36
Full-time	7	46.7	9	60		53.3		
Part-time	6	40	2	13.3		26.7		
Studying	1	6.7	3	20		13.3		
Unemployed	1	6.7	1	6.7		6.7		
Ethnicity							7.6 (7)	0.37
European/European descent	15	100	12	80	27	90		
Indian	-	-	2	13.3	2	6.7		
Chinese	-	-	1	6.7	1	3.3		
First language							**1 (2)**	0.6
English	15	100	14	93.3	29	96.7		
Other			1	6.7	1	3.3		

^1^*n* = 1 missing (unrecorded)

Demographic and characteristics are presented in Table 1 as a function of the total sample, and per treatment group. The final sample used for analysis comprised of equal numbers of males and females (*n* = 15 each). Overall, the sample had a mean age of 31.57 (SD±6.84) years, mean height of 174.76 cm (SD±9.61) and weight of 72.66 kg (SD±18.47). Most participants had a tertiary degree (63.3%), were employed full-time (53.7%) and spoke English as their first language (96.7%). No statistically significant group differences were observed in terms of these key demographic characteristics (all *p* > 0.05). One participant’s highest education level was left unrecorded due to a documenting error at baseline.

### Clinical outcomes

#### Neurocognitive performance (Online Resource 2–5)

There was a significant main effect of time for *simple reaction time* [F_(2,55.50)_ = 4.20, *p* = 0.02]. *Simple reaction time* showed a statistically significant decrease from after 1-week of dosing to after 2-weeks of dosing at trial completion (Online Resource 2) for the overall sample including both treatments (V3-V4 mean difference = -10.47, SE 3.84, *p* = 0.025) with no significant differences between treatments noted at any timepoint (Online Resource 2).

No main effect of time was observed for *numeric working memory accuracy* [F_(2,56)_ = 3.17, *p* = 0.05] (Online Resource 3) or *picture recognition accuracy* [F_(2, 48.31)_ = 2.91, *p* = 0.06] (Online Resource 5). No interaction of group and time was noted for *word recognition accuracy* [F_(2,56)_ = 3.09, *p* = 0.05] (Online Resource 5).

No other statistically significant effects for group, time or the interaction of Group and Time were observed (all *p* > 0.05) (Online Resources 2–5).

#### Subjective state effect outcomes (daily VAS) (Fig. 2)

Analyses revealed significant main effects for group [F_(1, 27.91)_ = 5.94, *p* = 0.021], time [F_(2, 565.79)_ = 13.35, *p* < 0.001] and the interaction of group and time [F_(2, 565.79)_ = 8.96, *p* < 0.001] were observed for *calm-excited*. Relative to placebo, those receiving CBD reported greater calmness/less excitement after 1-week of dosing with sustained effects observed after 2-weeks of dosing at trial completion (V4 mean difference = -1.46, SE 0.49, *p* = 0.005, [CI -2.45, -0.48], *d* = 0.99).

Main effects for group [F_(1, 28.07)_ = 9.57, *p* = 0.004], time [F_(2, 566.85)_ = 12.98, *p* < 0.001] and the interaction of group and time [F_(2, 566.84)_ = 5.33, *p* = 0.005] were noted for *clearheaded-muzzy*. Statistically significant treatment differences were observed, with those in the CBD group reporting improved clear-headedness/less muzziness compared to placebo after 1-week of dosing with sustained improvements observed after 2-weeks of dosing at trial completion (V4 mean difference = -1.74, SE 0.46, *p* < 0.001, [CI -2.65, -0.82], *d* = 0.96).

*Coordinated-clumsy* showed main effects for group [F_(1, 27.93)_ = 5.84, *p* = 0.022 ], time [F_(2, 564.74)_ = 5.26, *p* = 0.005] and the interaction of group and time [F_(2, 564.733)_ = 3.85, *p* = 0.022]. Compared to placebo, those receiving CBD reported improved coordination/less clumsiness after 1-week of dosing with sustained effects observed after 2-weeks of dosing at trial completion (V4 mean difference = -1.65, SE 0.56, *p* = 0.006, [CI -2.78, -0.51, *d* = 1.01).

There were observed main effects for group [F_(1,27.57)_ = 7.88, *p* = 0.009] and time [F_(2, 565.96)_ = 19.46, *p* < 0.001] for *happy-sad*. Post hoc analyses showed those receiving CBD reported greater happiness/less sadness than placebo throughout the whole trial period (V2, V3) (all *p* < 0.05) including after 2-weeks of dosing at trial completion (V4 mean difference= -1.42, SE 0.42, *p* = 0.002, [CI -2.27, -0.58], *d* = 1.00).

*Alert-drowsy* showed main effects for group [F_(1, 28)_ = 4.49, *p* = 0.043] and time [F_(2,566.79)_ = 17.19, *p* < 0.001]. Relative to placebo, those in the CBD group reported improved alertness/less drowsiness after 1-week of dosing and 2-weeks of dosing at trial conclusion (V4 mean difference = -1.26, SE 0.49, *p* = 0.014, [CI -2.24, -0.27], *d* = 0.65).

Main effects for group [F_(1, 28.12)_ = 7.02, *p* = 0.013] and time [F_(2, 566.08)_ = 8.79, *p* < 0.001] were noted for *energy-lethargy*. Post hoc analyses showed statistically significant differences between treatments throughout the whole trial period (V2, V3 and V4). Those receiving CBD reporting higher energy/less lethargy compared to placebo at all timepoints including trial conclusion (V4 mean difference = -1.13, SE 0.44, *p* = 0.0013, [CI -2, -0.25], *d* = 0.67).

*No appetite-ravenous* showed main effect for group [F_(1, 28.01)_ = 5.39, *p* = 0.028]. Post hoc analyses revealed the CBD group reported lower appetite/being less ravenous compared to placebo at the end of the placebo run-in period (V2 mean difference = -1.38, SE 0.58, [CI -2.56, -0.19, *p* = 0.024]) and after 2-weeks of dosing at trial conclusion (V4 mean difference = -1.44, SE 0.58, *p* = 0.018, [CI -2.63, -0.26], *d* = 0.78), with no statistically significant difference observed between treatments after 1-week of dosing (*p* > 0.05).

Feelings of *no fatigue-very fatigued* exhibited a main effect for time [F_(2,566.36)_ = 13.06, *p* < 0.001] with the CBD group reporting less fatigue compared to those in the placebo condition; however, this was not statistically significant at trial conclusion (*p* = 0.21).

Despite main effects for group and/or time for scales of *happy-sad*,* alert-drowsy*,* energy-lethargy*,* no appetite-ravenous* and *no fatigue-very fatigued*, no interaction effects of group and time were noted (all *p* > 0.05).

#### Side effects (daily log) (Online Resource 6)

The most frequent side effect reported over the trial period was *dry mouth* (51.67% of overall occurrences). Those who received CBD reported significantly more instances of dry mouth compared to those receiving placebo (*p* = 0.003, [CI-0.5, -0.9], *d* = 0.3), with a total of 25 occurrences reported by four participants in the CBD group, and six occurrences among three participants in the placebo group. Other reported side effects included nausea (16.67%), light headedness (13.33%), diarrhoea (11.67%) and dizziness (6.67%). Groups did not differ at any time in terms of reported instances of *nausea* (*p* = 0.13, [CI-0.3, 0.4], *d* = 0.2), *light-headedness* (*p* = 0.08, [CI -0.3, 0.5], *d* = 0.2), *diarrhoea* (*p* = 0.16, [CI -0.5, 0.3], *d* = 0.2) or *dizziness* (*p =* 0.41, [CI -0.3, 0.1], *d* = 0.1).

## Discussion

Outcomes from this pilot trial suggested nightly supplementation with 150 mg of CBD does not benefit daytime cognitive performance in people with primary insomnia when compared to placebo. Subjective states remained unimpaired as noted with sustained baseline ratings of calmness, clear headedness and co-ordination throughout the trial. There were negligible and transient side effects reported throughout the trial period. Overall, CBD may have some benefits for subjective mood over placebo; however, additional, larger, randomized controlled studies are required to more definitely determine these effects in populations with moderate-severe primary insomnia.

In studies assessing CBD effects on cognitive performance, limited improvements in tasks involving attentional switching, verbal learning, and memory in people who use cannabis (Solowij et al. 2018) and selective attention capacity, processing speed (Hallak et al. 2010), motor speed and executive functioning in people with schizophrenia had been attributed to its neuroprotective effects (Solowij et al. 2018), in addition to suggested linkages with its anxiolytic effects via the regulation of hypothalamic-pituitary-adrenal axis hyperarousal (Narayan et al. 2022). These effects were observed after both long-term administration (Solowij et al. 2018) and single, acute doses (Hallak et al. 2010). It is therefore unknown if these mechanisms were affected based on present cognitive performance outcomes under current study conditions or what effect the current dose size and frequency might have had. The absence of statistical differences between CBD and placebo groups throughout the trial period, including the start of the placebo run-in week suggest no discernible treatment effects of CBD on performance under this specific study design, methodology and timeframe. The timepoints at which improvements occurred for limited outcomes (simple reaction time and numeric working memory accuracy for new stimuli and reaction time) suggest the presence of a learning effect, as speed and accuracy of task completion have previously been observed to increase with repetition (Tao et al. 2019). Comprehensive systematic reviews and meta-analyses reported small to moderate cognitive impairments in the general domains of attention, memory, concentration and executive function (Ballesio et al. 2019; Brownlow et al. 2020; Fortier-Brochu et al. 2012; Wardle-Pinkston et al. 2019). Yet, key limitations emerged from the utilization of cognitive tasks established and validated for neurological conditions known to present major cognitive deficits such as traumatic brain injuries (Fortier-Brochu et al. 2012; Brownlow et al. 2020). This highlighted issues regarding task sensitivity and the detection of subtle cognitive impairments commonly reported in insomnia (Fortier-Brochu et al. 2012). Resultantly, the sensitivity of current validated cognitive tasks to detect subtle impairments is uncertain and warranted necessary validation in samples with insomnia. Moreso, the comparisons of different tasks measuring the same domain across studies further presented a significant limitation in accurately characterizing cognitive performance in insomnia (Fortier-Brochu et al. 2012). Comparable demographic characteristics in each group suggest that other, otherwise unmeasured factors, may have played a role in the overall sample improvements seen in these domains. Importantly, while we did not observe an improvement in performance, a lack of definitively impairing effects produced by continuous CBD dosing demonstrates a decided advantage over frequently prescribed drugs for insomnia, which are known to impair performance over numerous cognitive domains with both acute and chronic administration (Stewart 2005), given past observations of CBD’s potential sleep and mood effects (Narayan et al. 2022; Ranum et al. 2022; Suraev et al. 2020). It must be noted that the time to the maximum measured plasma concentration (T_max_) for sublingual CBD is anywhere between 0 and 4 h (Millar et al. 2018). As cognitive tests were often completed over 8 h following the previous night’s dose, we acknowledge that this is unlikely to align to the expected the T_max_ of CBD (Millar et al. 2018). Nonetheless, we argue that these timelines appropriately align with typical schedules for consumption of this treatment as a sleep remedy, and for assessing potential impacts on daytime functional performance. As the half-life of CBD can vary considerably depending on dose and administration route, any potential treatment hangover effects that could potentially diminish performance were not observed under present conditions (Millar et al. 2018; Nicholson et al. 2004).

Subjective states outcomes demonstrated that at baseline, the CBD group reported greater energy, happiness and lower appetite and therefore, changes to these scales described over the course of the trial indicated that these were not a result of receiving the active treatment. Similarly, despite increased feelings of alertness after being administered CBD, without interaction effects, it is most likely that other factors including changes in daily routines caused by trial participation may have contributed to this effect over time (Perlis et al. 2005). It also noted that feelings of lethargy, sadness, drowsiness or daily appetite did not increase for participants after receiving CBD as measured. Contrastingly, statistically significant interaction effects demonstrating differences between groups during the 2-weeks of active treatment for feelings of calm, clear-headedness and coordination suggested possible treatment effects of CBD. Previous neuroimaging assessments in clinical populations have shown acute CBD doses produced significant changes in the modulation of functional networks and resting limbic activity in conditions including people with psychosis (O’Neill et al. 2021) and generalized social anxiety disorder (Crippa et al. 2011), potentially reflecting its mechanisms for therapeutic benefits in emotional processing (Batalla et al. 2021) over cognitive performance. In contrast, unchanged mood was noted in a sample with schizophrenia when dosing with 600 mg CBD (Boggs et al. 2018), further suggesting these mood effects were dose-dependent and highlight the importance of dose size and frequency for therapeutic effects (Linares et al. 2019; Zuardi et al. 2017; Narayan et al. 2022). Though no consensus exists on beneficial therapeutic doses for mood, higher acute doses (300 mg-400 mg) than those used in this study (Zuardi et al. 2017; Linares et al. 2019) have previously shown anxiolytic effects suggesting the 150 mg was too small for any significant mood improvements. In addition to this, the elevated baseline feelings of energy and happiness in the CBD group suggested the presence of a positive assessment bias as a result of various group differences including lifestyle changes and environmental factors, that may have influenced observed outcomes at the end of the trial. This further signalled the need for results concerning the treatment effects of CBD to be interpreted cautiously.

To our knowledge, this trial was the first to explore effect of CBD on cognitive performance in a population with primary insomnia, further adding to the narrow breadth of research available on cannabinoid medicine, primary insomnia, and cognitive performance. Due to the pilot nature of this trial and the small sample size utilized, there are some notable limitations in the generalizability of our findings. Specifically, primary insomnia was analysed as a homogenous condition and may have resulted in phenotype-specific differences in cognitive performance outcomes between treatments being undetected (Ballesio et al. 2019; Gencarelli et al. 2020). The present results should therefore be considered in the context of these limitations when being generalized to wider groups of individuals with primary insomnia, and more specifically, those using CBD medicinally. The criteria for identifying placebo responders were also purposefully stringent to identify only those participants with the strongest improvements to baseline sleep over the placebo run-in period. Including placebo responders may unintentionally distort results, interfering with the potential to identify treatment effects (Dumitrescu et al. 2019)., and this approach may also inadvertently reduce the generalizability of these findings to the broader insomnia population (Kärppä et al. 2020). We also acknowledge that these exclusions may have resulted in an overestimation of treatment efficacy (Trivedi and Rush 1994). Taking the limitations discussed into consideration, it is stressed that the outcomes of this trial must be interpreted cautiously, with future trials necessary to corroborate outcomes. Despite these limitations, this trial effectively managed confounding factors through robust methodological processes of randomization, double-blinding and strict screening, and exclusion procedures. Nonetheless, we acknowledge that unmeasured factors inherent in the design and implementation of therapeutic trials designed to address sleep disturbance, including changes to sleep hygiene (Stepanski and Wyatt 2003) and overall the influence of trial conditions (Perlis et al. 2005) may have affected outcomes. Several measures were implemented to uphold participant adherence and consistency. These included verbal checks at the beginning of each visit for any cannabis use, excluding people with high caffeine consumption during screening and daily tracking of deviations from participants’ routines including exercise, alcohol consumption and hangovers logged via self-report in sleep diaries. Beyond these measures, adherence monitoring was limited with testing scheduling consistency challenged by changing participant availabilities falling outside the expected start times (9:30am − 12pm). Additionally, it is uncertain how effective blinding was without its ongoing explicit assessment throughout the 3-week trial period. Therefore, high quality crossover studies are required to confirm results and are recommended to analyse differences in primary insomnia based on objective and subjective phenotypes to determine differences between treatment groups when testing the efficacy of CBD. Future studies are thus recommended to explore and compare higher and lower acute CBD doses taken daily for varying treatment periods in the context of these phenotypes whilst utilizing a larger sample size. The pharmacokinetic profile of CBD is also recommended to be explored within these contexts to highlight patterns of metabolism, distribution, and elimination in relation to potential therapeutic benefits (Britch et al. 2021). Use of a crossover design may aid in mitigating participant variations in cognitive ability (Helle et al. 2019) and clarifying neurocognitive differences within those with insomnia whilst assessing the efficacy of CBD treatment effects for this group; however, the potential for introducing further confounding learning effects should be considered when using this design.

With growing interest in the clinical and therapeutic profile of cannabinoid products, these results support the preservation of daily cognitive functioning, calmness, clear-headedness and co-ordination over two weeks of nightly consumption of 150 mg of CBD when used to manage symptoms of self-reported primary insomnia. It additionally corroborates the minimal and transient negative side effects that past studies have reported with short term use and recommends an increased need to explore side effects of its longitudinal use and drug interactions (Huestis et al. 2019). To better clarify the magnitude of these effects, larger trials are urgently needed to corroborate present findings and differentiate between treatment effects and biases perpetuated by limitations in trial design, measures and participants to help establish robust therapeutic guidelines as over the counter CBD availability increases.

## Electronic supplementary material

Below is the link to the electronic supplementary material.

Supplementary Material 1

## Data Availability

Data supporting these findings are available within this article.
