# Supplementary material for: The effect of nightly use of 150 mg cannabidiol on daytime neurocognitive performance in primary insomnia: a randomized controlled pilot trial
Source: Psychopharmacology (Berl). 2024 Aug 17;242(2):297–308. doi: 10.1007/s00213-024-06674-x (PMC11774964; doi:10.1007/s00213-024-06674-x)
Supplement: Supplementary file 1 — Supplementary Material 1 [file 213_2024_6674_MOESM1_ESM.docx]

**Supplementary Information**

**List of Supplementary Tables and Figures:**

- **Online Resource 1: Participant Flow Diagram -** Figure of adapted CONSORT participant flow diagram showing recruitment at each trial phase
- **Online Resource 2: Simple reaction time, complex reaction time and digit vigilance outcomes -** Table of raw means, standard deviation for both treatments at each time point with interaction terms
- **Online Resource 3: Numeric and spatial working memory outcomes -** Table of raw means, standard deviation for both treatments at each time point with interaction terms
- **Online Resource 4: Immediate and delayed recall outcomes -** Table of raw means, standard deviation for both treatments at each time point with interaction terms
- **Online Resource 5: Word and picture recognition outcomes -** Table of raw means, standard deviation for both treatments at each time point with interaction terms
- **Online Resource 6: Side Effects -** Stacked bar graph of total sides effects (%) reported by CBD and placebo treatment groups over the trial period.

**Online Resource 1:** Participant Flow Diagram

Assessed for eligibility (n=164)

Excluded (n=76)

- ISI (n=30)
- Medical condition/medication (n=27)
- Commitment (n=6)
- Age (n=4)
- Other (n=9)

Recruited (n=88)

Withdrew (n=18)

Excluded at V0 (n=18)

Reasons:

- BAI/BDI (n=15)
- Other (n=3)

Enrolment

Allocated to CBD (n=18)

Allocated to Placebo (n=16)

Lost to follow-up

V3: adverse event (n=1)

Analysed (n=15)

Recruited to mitigate attrition:

excluded from analysis (n=2)

Allocation

Lost to follow-up

V3: withdrew (n=1)

Follow-up

Analysed (n=15)

Analysis

Randomized (n=34)

V1 (attended n=44)

Withdrew (n=1)

Excluded at V2 (n=9)

Reasons:

- Placebo responders (n=8)
- Adverse event (n=1)

**Online Resource 1:** Figure of adapted CONSORT participant flow diagram showing recruitment at each trial phase

**Online Resource 2: Simple reaction time, complex reaction time and digit vigilance outcomes**

| **TASK** | **TREATMENT** | **PLACEBO RUN-IN START** | **AFTER 1-WEEK DOSING** | **AFTER 2-WEEKS DOSING** | **F-VALUE** | **P-VALUE** |
| --- | --- | --- | --- | --- | --- | --- |
|  |  | **mean**  **(SD)** | | |  |  |
| **REACTION TIME** | | | | | | |
| Simple median (ms) | *CBD* | 296.03 (29.16) | 289.47 ^#^ (35.75) | 301.30 ^#^ (31.10) | 0.04_(1, 28.2)_ | 0.84^-^ |
|  | *PLA* | 292.17 (33.54) | 289.30 ^#^ (31.66) | 298.40 ^#^ (26.95) |  |  |
| Complex accuracy (%) | *CBD* | 95.07 (3.85) | 94.93 (3.99) | 93.87 (4.24) | 3.25_(1, 28)_ | 0.08 |
|  | *PLA* | 95.87 (3.07) | 96.67 (2.58) | 96.4 (2.75) |  |  |
| Complex mean (ms) | *CBD* | 453.03 (64.75) | 454.10 (65.52) | 468.53 (66.60) | 0.25_(1, 28.36)_ | 0.62 |
|  | *PLA* | 446.90 (51.66) | 444.27 (48.33) | 453.63 (61.80) |  |  |
| **DIGIT VIGILANCE** | | | | | | |
| Accuracy (%) | *CBD* | 94.93 (5.56) | 95.38 (5.24) | 96.29 (4.42) | 2.72_(1, 30.52)_ | 0.11 |
|  | *PLA* | 97.48 (3.93) | 97.46 (3.03) | 97.19 (4.16) |  |  |
| Reaction time (ms) | *CBD* | 437.25 (52.44) | 435.69 (51.30) | 454.33 (49.17) | 1.45_(1, 28)_ | 0.24 |
|  | *PLA* | 426.29 (38.15) | 422.16 (28.41) | 427 (30.57) |  |  |
| False alarms | *CBD* | 0.07 (0.26) | 0.07 (0.26) | 0.00 (0.00) | 0.66_(1, 28)_ | 0.42 |
|  | *PLA* | 0.20 (0.56) | 0.07 (0.26) | 0.07 (0.26) |  |  |

Note: ms = milliseconds; % = percentage; *CBD* = cannabidiol; *PLA* = placebo;

^#^ = indicates significant within-treatment change over time, *p*<0.05;

^-^ =main effect of time, *p*<0.05; ^--^ =main effect of time, *p*<0.001;

Online Resource 2: Table of raw means, standard deviation for both treatments at each time point with interaction terms

**Online Resource 3: Numeric and spatial working memory outcomes**

| **TASK** | **TREATMENT** | **PLACEBO RUN-IN START** | **AFTER 1-WEEK DOSING** | **AFTER 2-WEEKS DOSING** | **F-VALUE** | **P-VALUE** |
| --- | --- | --- | --- | --- | --- | --- |
|  |  | **Mean (SD)** | | |  |  |
| **NUMERIC WORKING MEMORY** | | | | | | |
| Accuracy (%) | *CBD* | 93.11 ^#^  (7.32) | 95.26 ^#^ (3.91) | 94.74 (4.17) | 0.26_(1, 28)_ | 0.62 |
|  | *PLA* | 92.00 ^#^  (11.68) | 95.04 ^#^ (7.30) | 92.15 (9.52) |  |  |
| Reaction time mean (ms) | *CBD* | 733.39 (169.90) | 688.51 (117.48) | 729.38 (177.86) | 0.23_(1,28)_ | 0.63 |
|  | *PLA* | 725.06 (174.50) | 671.99 (164.83) | 682.71 (122.28) |  |  |
| **SPATIAL WORKING MEMORY** | | | | | | |
| Accuracy (%) | *CBD* | 96.13  (2.91) | 96.25 (4.60) | 93.75 (8.84) | 0.01_(1, 28)_ | 0.91 |
|  | *PLA* | 94.67  (4.89) | 96.63 (2.87) | 93.88 (6.53) |  |  |
| Reaction time mean (ms) | *CBD* | 700.31  (117.68) | 698.13 (120.01) | 691.93 (139.15) | 1.69_(1, 28)_ | 0.20 |
|  | *PLA* | 679.75 (168.51) | 634.05 (139.61) | 610.36 (121.73) |  |  |

Note: ms=milliseconds; % = percentage; *CBD* = cannabidiol; *PLA* = placebo;

^#^ = indicates significant within-treatment change over time, *p*<0.05.

Online Resource 3: Table of raw means, standard deviation for both treatments at each time point with interaction terms

**Online Resource 4: Immediate and delayed recall outcomes**

| **TASK** | **TREATMENT** | **PLACEBO RUN-IN START** | **AFTER 1-WEEK DOSING** | **AFTER 2-WEEKS DOSING** | **F-VALUE** | **P-VALUE** |
| --- | --- | --- | --- | --- | --- | --- |
|  |  | **Mean (SD)** | | |  |  |
| **IMMEDIATE RECALL** | | | | | | |
| Words correctly recalled | *CBD* | 7.33  (2.77) | 7.67 (3.11) | 8.40 (3.02) | 0.06_(1,28)_ | 0.81 |
|  | *PLA* | 7.27  (2.37) | 7.67 (3.09) | 7.80 (2.43) |  |  |
| Accuracy (%) | *CBD* | 48.89 (18.46) | 51.11 (20.73) | 56.00 (20.13) | 0.06_(1,28)_ | 0.81 |
|  | *PLA* | 48.44 (15.83) | 51.11 (20.57) | 52.00 (16.17) |  |  |
| Recall errors | *CBD* | 0.47  (0.52) | 0.60 (0.63) | 0.27 (0.46) | 0.01_(1, 37.78)_ | 0.95 |
|  | *PLA* | 0.40  (0.83) | 0.67 (1.11) | 0.53 (0.64) |  |  |
| **DELAYED RECALL** | | | | | | |
| Words correctly recalled | *CBD* | 6.53  (3.18) | 7.13 (4.12) | 6.93 (3.49) | 0.14_(1,28)_ | 0.71 |
|  | *PLA* | 5.93  (3.31) | 6.20 (3.26) | 7.13 (3.23) |  |  |
| Accuracy (%) | *CBD* | 43.55 (21.21) | 47.56 (27.47) | 46.22 (23.30) | 0.14_(1,28)_ | 0.71 |
|  | *PLA* | 39.55 (22.03) | 41.33 (21.71) | 47.56 (21.51) |  |  |
| Recall errors | *CBD* | 0.73  (0.88) | 0.67 (0.90) | 0.33 (0.49) | 0.53_(1,31.68)_ | 0.47 |
|  | *PLA* | 1.33  (0.53) | 0.87 (1.19) | 0.53 (0.92) |  |  |

Note: ms = milliseconds; % = percentage; *CBD* = cannabidiol; *PLA* = placebo.

Online Resource 4: Table of raw means, standard deviation for both treatments at each time point with interaction terms

**Online Resource 5: Word and picture recognition outcomes**

| **TASK** | **TREATMENT** | **PLACEBO RUN-IN START** | **AFTER 1-WEEK DOSING** | **AFTER 2-WEEKS DOSING** | **F-VALUE** | **P-VALUE** |
| --- | --- | --- | --- | --- | --- | --- |
|  |  | **Mean (SD)** | | |  |  |
| **WORD RECOGNITION** | | | | | | |
| Accuracy (%) | *CBD* | 80.67 # (12.74) | 85.33 # (13.08) | 84.67  (12.20) | 0.09_(1,28)_ | 0.76 |
|  | *PLA* | 83.78 (10.07) | 80  (13.63) | 84.22  (10.42) |  |  |
| Reaction time mean (ms) | *CBD* | 816.52 (147.71) | 774.08 (89.84) | 777.56 (145.91) | 0.79_(1, 25.87)_ | 0.38 |
|  | *PLA* | 797.61 (133.49) | 733.21 (82.26) | 747.12  (84.31) |  |  |
| **PICTURE RECOGNITION** | | | | | | |
| Accuracy (%) | *CBD* | 86.17 (13.33) | 86.33  (11.76) | 86.50  (10.12) | 0.00_(1,26.67)_ | 0.97 |
|  | *PLA* | 82  (13.98) | 79.11# (16.19) | 87.32 #  (9.73) |  |  |
| Reaction time mean (ms) | *CBD* | 890.13 (128.28) | 900.99 (132.11) | 918.63 (213.40) | 0.13_(1, 28)_ | 0.72 |
|  | *PLA* | 880.57 (167.41) | 956.12 (132.38) | 896.41 (131.27) |  |  |

Note: ms=milliseconds; % = percentage; original = original stimuli; new = new stimuli; *CBD* = cannabidiol; *PLA* = placebo;

# = indicates significant within-treatment change over time, *p*<0.05.

Online Resource 5: Table of raw means, standard deviation for both treatments at each time point with interaction terms

**Online Resource 6:** Side Effects

**Online Resource 6:** Stacked bar graph of total side effects (%) reported by CBD and placebo treatment groups over the trial period
